# Targeting receptor for activated C kinase 1 with a small molecule induces mitotic catastrophe and suppresses lipid metabolic reprogramming in hepatocellular carcinoma

**DOI:** 10.1002/ctm2.70460

**Published:** 2025-08-20

**Authors:** Longyan Wang, Peng Tan, Fei Wang, Huiming Huang, Xuejiao Wei, Zhuguo Wang, Xinyu Qiu, Yufeng Gao, Ruoxin Zhang, Pengfei Tu, Jun Li, Zhongdong Hu

**Affiliations:** ^1^ Department of Pharmacology of Traditional Chinese Medicine, School of Chinese Materia Medica Beijing University of Chinese Medicine Beijing China; ^2^ Modern Research Centre for Traditional Chinese Medicine Beijing Research Institute of Chinese Medicine Beijing University of Chinese Medicine Beijing China

1

Dear Editor,

Hepatocellular carcinoma (HCC) is a widespread, life‐threatening malignancy. The initial symptoms of HCC are often inconspicuous, but the disease progresses swiftly, readily metastasising and disseminating, thus reaching a high mortality rate.^1^ Traditional Chinese medicine (TCM) is especially advantageous in treating cancer and offers promising avenues in terms of discovering the active compounds of TCM that can be used in cancer therapy.^2^

The resin *Resina Draconis* is obtained through the ethanol extraction of fatty wood from *Dracaena cochinchinensis* (Lour.) S.C. Chen. Recent pharmacological studies have confirmed its efficacy in terms of exhibiting anti‐inflammatory, analgesic, hypolipidemic and antitumor properties.^3^ The natural flavonoid (*R*)–7,3′‐dihydroxy‐4′‐methoxy‐8‐methylflavane (DHMMF) was obtained through the ethyl acetate fraction of *R. Draconis* in our laboratory (Figure 1A). Preliminary findings indicated that DHMMF exhibits significant in vitro and in vivo activity against HCC.^4^ However, the mechanisms through which DHMMF exerts its anti‐HCC effect remain to be fully elucidated to date.

**FIGURE 1:**
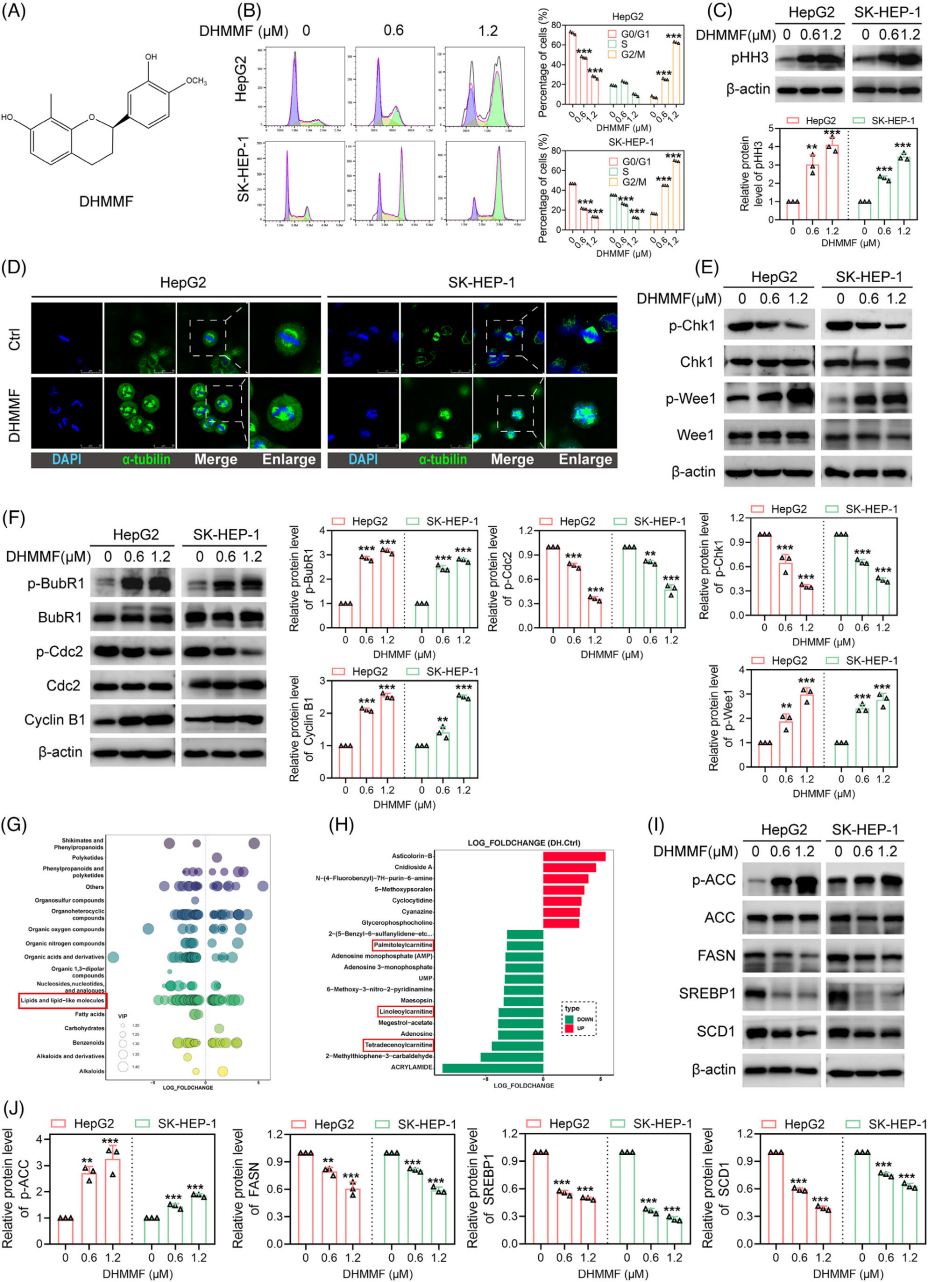
(*R*)–7,3′‐dihydroxy‐4′‐methoxy‐8‐methylflavane (DHMMF) induced mitotic catastrophe and modulated lipid metabolism in human hepatocellular carcinoma (HCC) cells. (A) Chemical formula of DHMMF. (B) Human HCC cells were subjected to 24 h of treatment with DHMMF at concentrations of 0, 0.6 and 1.2 µM, after which the cell cycle distribution was detected using flow cytometry (*n* = 3). (C) Human HCC cells were subjected to 24 h of treatment with DHMMF at concentrations of 0, 0.6 and 1.2 µM. The pHH3 protein level was subsequently measured using western blotting (*n* = 3). (D) Human HCC cells were subjected to 24 h of treatment with DHMMF at concentrations of 0, 0.6 and 1.2 µM, and then, α‐tubulin immunofluorescence staining was used to observe the spindle morphology during mitosis (*n* = 3); scale bar: 25 µm. (E) Human HCC cells were treated with 0, 0.6 or 1.2 µM of DHMMF for 24 h. Later, the levels of G2/M phase checkpoint‐related protein were detected using western blotting (*n* = 3). (F) Human HCC cells were subjected to 24 h of treatment with DHMMF at concentrations of 0, 0.6 and 1.2 µM. Thereafter, BubR1, Cdc2 and Cyclin B1 protein levels were measured using western blotting (*n* = 3). (G and H) Classification of the major metabolic changes in the human HCC cells before and after 24 h of DHMMF treatment using nontargeted metabolomics. (I and J) Human HCC cells were treated with 0, 0.6 or 1.2 µM of DHMMF for 24 h. Protein expression levels related to lipid metabolism were subsequently measured using western blotting (*n* = 3). ^**^ *p* < 0.01 and ^***^ *p* < 0.001.

Receptor for activated C kinase 1 (RACK1) serves as a versatile scaffolding protein with multifaceted regulatory roles in cell proliferation, gene transcription and protein synthesis.^5^ An increase in its expression promotes the growth of human HCC cells.^6^ Silencing RACK1 was found to inhibit the proliferation of human HCC MHCC97‐H cells.^7^ In HCC cells, RACK1 potentiates the translation of oncogenes and the glycosylation status of RACK1 was found to increase in pathological tissue samples derived from HCC patients.^8^ These findings indicated the potential of RACK1 as a therapeutic target in HCC.

As shown in Figure S1A,B, DHMMF strongly suppressed the proliferation of human HCC cells (HepG2 and SK‐HEP‐1) in this study. Flow cytometry analysis further revealed that DHMMF induced cell cycle arrest at the G2/M phase in these cells (Figure 1B). Phospho‐histone H3 (pHH3), which is considered a definitive marker for the M phase of the cell cycle,^9^ was significantly upregulated in both cell lines after treatment with DHMMF (Figure 1C). Consequently, it was inferred that DHMMF effectively induces HCC cell cycle arrest at the M phase. Mitotic catastrophe is a cell death phenomenon that stems from mitotic deregulation, which is generally triggered by an abnormal spindle assembly and is frequently accompanied by spindle multipolarity, multinucleation and other related phenomena.^10^ Staining performed for α‐tubulin provides insights into the morphological alterations of the spindle during cell mitosis. In this study, specifically, after 24 h of treatment with DHMMF, multipolar spindles emerged in both cell lines (Figure 1D), suggesting that DHMMF induces mitotic catastrophe in human HCC cells.

Both DNA damage induction and inhibition of the functions of the G2/M checkpoint proteins reportedly elicit mitotic catastrophe in cells.^11^ According to the results of the comet assays conducted in this study, DHMMF administration resulted in tailing phenomena in the DNA electrophoresis images of the HCC cells (Figure S1C). DHMMF also increased the expression of the γH2AX protein in both cell lines (Figure S1D), confirming that DNA was damaged in these human HCC cells following DHMMF treatment. DNA damage triggers G2/M checkpoint activation for DNA damage repair, thus preventing the DNA‐damaged cells from progressing to mitosis.^12^, ^13^ As shown in Figure 1E, DHMMF treatment considerably increased the phosphorylation levels of Wee1 (Ser642) and decreased the phosphorylation levels of Chk1 (Ser317) in the human HCC cells, suggesting that DHMMF impairs the G2/M checkpoint functions in human HCC cells. When the G2/M checkpoint is inhibited, the DNA‐damaged cells prematurely enter mitosis, subsequently activating the spindle assembly checkpoint (SAC), resulting in extended M‐phase arrest and mitotic catastrophe.^14^, ^15^, ^16^ The Cyclin B‐Cdc2 complex has an important effect in terms of regulating the transition between the G2 and M phases.^17^ After entering the M phase, the complex becomes reactivated and drives the progression of mitosis, and at the end of mitosis, Cyclin B undergoes ubiquitination and degradation, leading to its exit from mitosis.^17^ Damaged chromosomes fail to align properly, thereby activating the spindle assembly checkpoint protein BubR1, which inhibits the activation of the anaphase‐promoting complex (APC/C) and its effect in terms of the ubiquitination of the substrate Cyclin B1, thereby preventing the transition from metaphase to anaphase during mitosis.^18^ In this study, as shown in Figure 1F, DHMMF administration considerably increased the BubR1 (Ser670) phosphorylation and Cyclin B1 protein levels while notably reducing the phosphorylation levels of Cdc2 (Tyr15) in human HCC cells. These findings indicated that DHMMF arrests the cell cycle at metaphase in human HCC cells. In summary, DHMMF can induce DNA damage in human HCC cells and inhibit the functions of the G2/M checkpoint proteins, thereby causing the DNA‐damaged cells to prematurely enter mitosis. This, in turn, activates the SAC, leading to M‐phase arrest and mitotic catastrophe.

Next, transcriptome sequencing was conducted for HepG2 cells before and after DHMMF treatment, which was followed by a comprehensive GO enrichment analysis of the obtained data. As shown in Figure S2A, DHMMF strongly regulated the metabolic processes in human HCC cells. In order to further assess the effects of DHMMF, an untargeted metabolomics analysis on HepG2 cells was performed before and after DHMMF treatment. The results (Figure S2B and Figure 1G) indicated that most of the cellular metabolites were downregulated after DHMMF treatment, with the differentially abundant metabolites enriched in lipids and lipid‐like small molecules. Among the top 20 metabolites with the most significant changes, most of the lipid molecules were downregulated (Figure 1H). Moreover, a substantial proportion of these differentially abundant metabolites were associated with the glycerophospholipid metabolism pathway (Figure S2C). Using a triglyceride (TG) detection kit, it was revealed that DHMMF treatment decreased the TG levels in both types of human HCC cells (Figure S2D). This study also investigated how DHMMF affects the lipid metabolism‐related proteins in human HCC cells. DHMMF administration was found to significantly suppress the expressions of SREBP1, FASN and SCD1 while concurrently increasing the phosphorylation levels of ACC in both HCC cell lines (Figure 1I,J). These findings suggested that DHMMF may inhibit lipid synthesis in human HCC cells by modulating the functions of SREBP1 and its downstream proteins ACC, FASN and SCD1, thus exerting anti‐HCC effects.

In order to further identify the direct target of DHMMF in HCC, chemical synthesis techniques were employed to introduce a biotin moiety at the 7‐hydroxyl position of DHMMF and thereby synthesising a biotin‐labelled probe (Figure 2A). The structure of the probe was confirmed using mass spectrometry and nuclear magnetic resonance analysis (Figure S3A and S3B). A target fishing experiment was performed. Proteins bound to the biotin‐labelled DHMMF were enriched using the biotin‐avidin system, followed by enzymatic digestion and mass spectrometric analysis. A biotin‐only control group was established to exclude the non‐specific binding (Figure 2B). After the mass spectrometry results were analysed and the interference from biotin was eliminated, 177 candidate proteins were obtained (Figure S3C). Scoring the reliability of these candidate proteins revealed the top 10 proteins (Figure S3D). A protein functional analysis of these candidate proteins prioritised RACK1 as a direct target protein of DHMMF. The results of the pull‐down immunoblotting assay revealed that the biotin‐labelled DHMMF could effectively “fish out” the RACK1 protein. When excess DHMMF molecules were added to compete for binding, the amount of RACK1 protein “fished out” by the biotin‐labelled DHMMF decreased (Figure 2C). The cell thermal shift assay results suggested that, relative to that of the control group, the degradation rate of the RACK1 protein co‐incubated with DHMMF decreased as the temperature increased, suggesting that DHMMF increased the thermal stability of RACK1 (Figure 2D). The drug affinity responsive target stability experiment results revealed that DHMMF increased the protease stability of RACK1 (Figure 2E). The SPR experiment results revealed that the dissociation constant between DHMMF and RACK1 was 3.52 × 10^−6^ M (Figure 2F), indicating a strong affinity between the two. The molecular docking experiments revealed that DHMMF could fit snugly into the cavity structure of the RACK1 protein, with a strong binding force and a binding energy of –7.8 kcal/mol. In addition, DHMMF formed hydrogen bonds with the RACK1 protein through the threonine positions 19 and 287, glutamine position 20, and arginine position 155 (Figure 2G). The Kaplan‐Meier database (http://kmplot.com) was used for analysis.^19^, ^20^ RACK1 levels within the tumour tissues of patients with liver cancer were determined. Compared to those in the non‐carcinoma samples, the RACK1 levels in liver cancer samples were increased (Figure 2H). The Kaplan‐Meier database revealed that high RACK1 expression in liver cancer patients was negatively associated with the overall survival, recurrence‐free survival and progression‐free survival (Figure 2I), demonstrating that RACK1 is a therapeutic target for liver cancer. Therefore, RACK1 may be a direct target through which DHMMF exerts its anti‐HCC effects.

**FIGURE 2:**
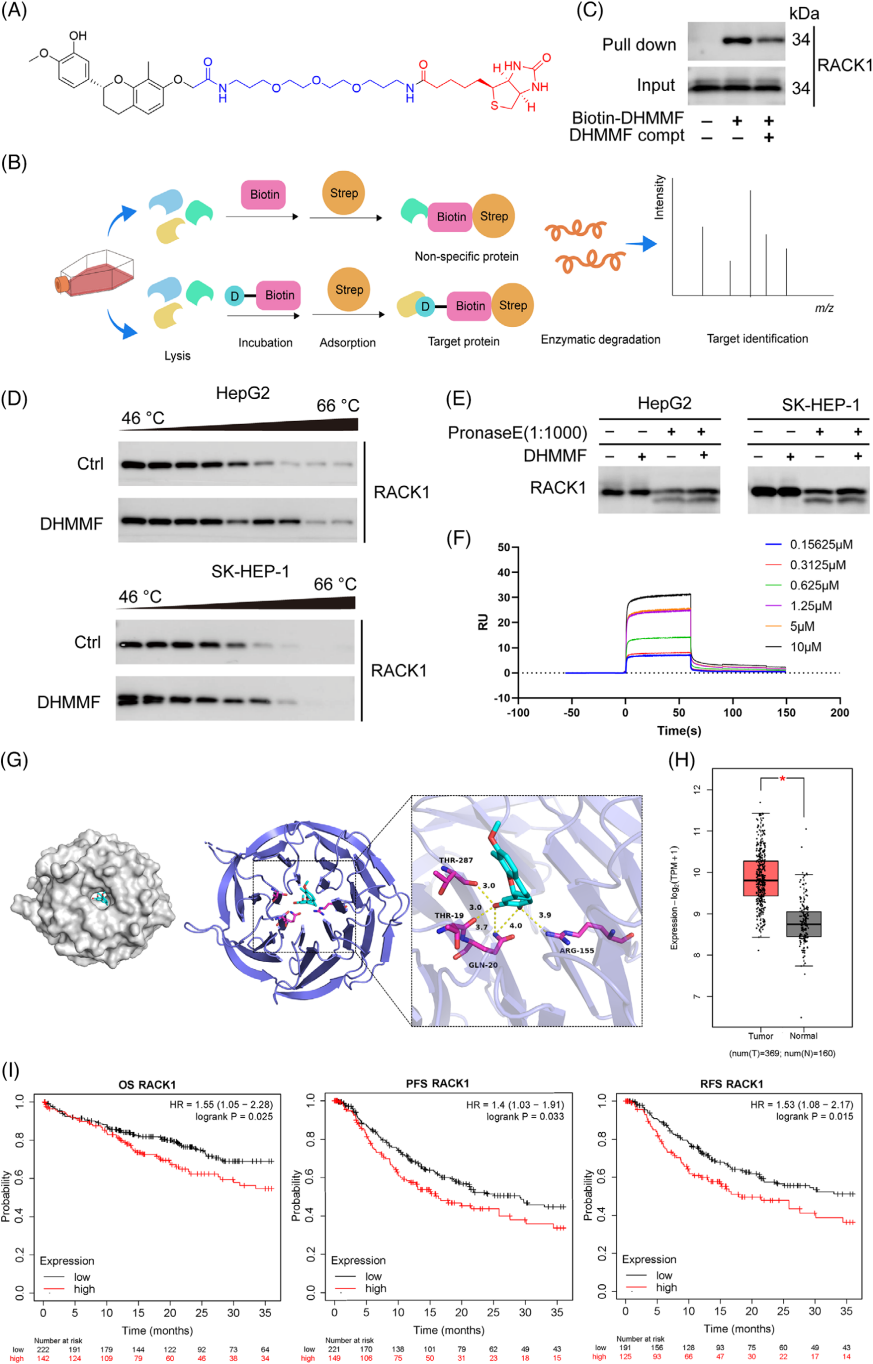
Receptor for activated C kinase 1 (RACK1) is a direct target of (*R*)–7,3′‐dihydroxy‐4′‐methoxy‐8‐methylflavane (DHMMF). (A) DHMMF was conjugated to biotin molecules through carbon chains. (B) An illustration of the DHMMF target pull‐down assay, in which the biotin group was used as a control and proteins bound to the biotinylated DHMMF were enriched using the biotin‐avidin system, followed by enzymatic digestion and mass spectrometry analysis. (C) HepG2 cells were pretreated with 100 µM DHMMF and incubated overnight at 4°C. After treatment, the treated and untreated lysates were incubated separately with 10 µM DHMMF and 50 µM biotin‐linked DHMMF in a reverse incubation manner. (D) After HepG2 and SK‐HEP‐1 cells were treated with 10 µM DHMMF for 2 h, the thermal stability of the RACK1 protein was assessed using a cell thermal shift assay (CETSA). (E) After both the cell lines were treated with 10 µM DHMMF for 2 h, the enzymatic degradation stability of the RACK1 protein was evaluated using the drug affinity responsive target stability (DARTS) assay. (F) SPR analysis revealed strong binding affinity between DHMMF and RACK1, with a KD of 3.52 × 10^−6^ M. (G) Molecular docking simulation revealed the three‐dimensional structure of the DHMMF‐RACK1 complex, with a binding energy of –7.8 kcal/mol. (H) RACK1 expression in the tumour tissues from liver cancer patients was determined using the Kaplan‐Meier plotter database. (I) The associations of RACK1 expression with the overall survival (OS), progression‐free survival (PFS) and recurrence‐free survival (RFS) in patients with liver cancer were assessed using the Kaplan‐Meier plotter database. ^*^ *p* < 0.05.

In order to determine the effect of RACK1 on the anti‐HCC activity of DHMMF, RNA interference technology was used to knock down RACK1 expression in human HCC cells. The subsequent quantitative real‐time polymerase chain reaction and western blotting experiments confirmed the successful knockdown of RACK1 expression in both human HCC cell lines (Figures 3A and 4B). The RACK1 knockdown was observed to decrease cell sensitivity to DHMMF treatment (Figure 3C), suggesting that RACK1 plays an important role in the ability of DHMMF to reduce human HCC cell viability. Silencing RACK1 also mitigated the G2/M phase arrest capacity of DHMMF in the HCC cells (Figure 3D). Additionally, knocking down RACK1 expression weakened the regulatory effect of DHMMF on the lipid metabolism‐related proteins in HCC cells (Figure 3E). Thus, RACK1 is intricately related to the mechanisms by which DHMMF regulates cell cycle arrest and lipid metabolism in human HCC cells.

**FIGURE 3:**
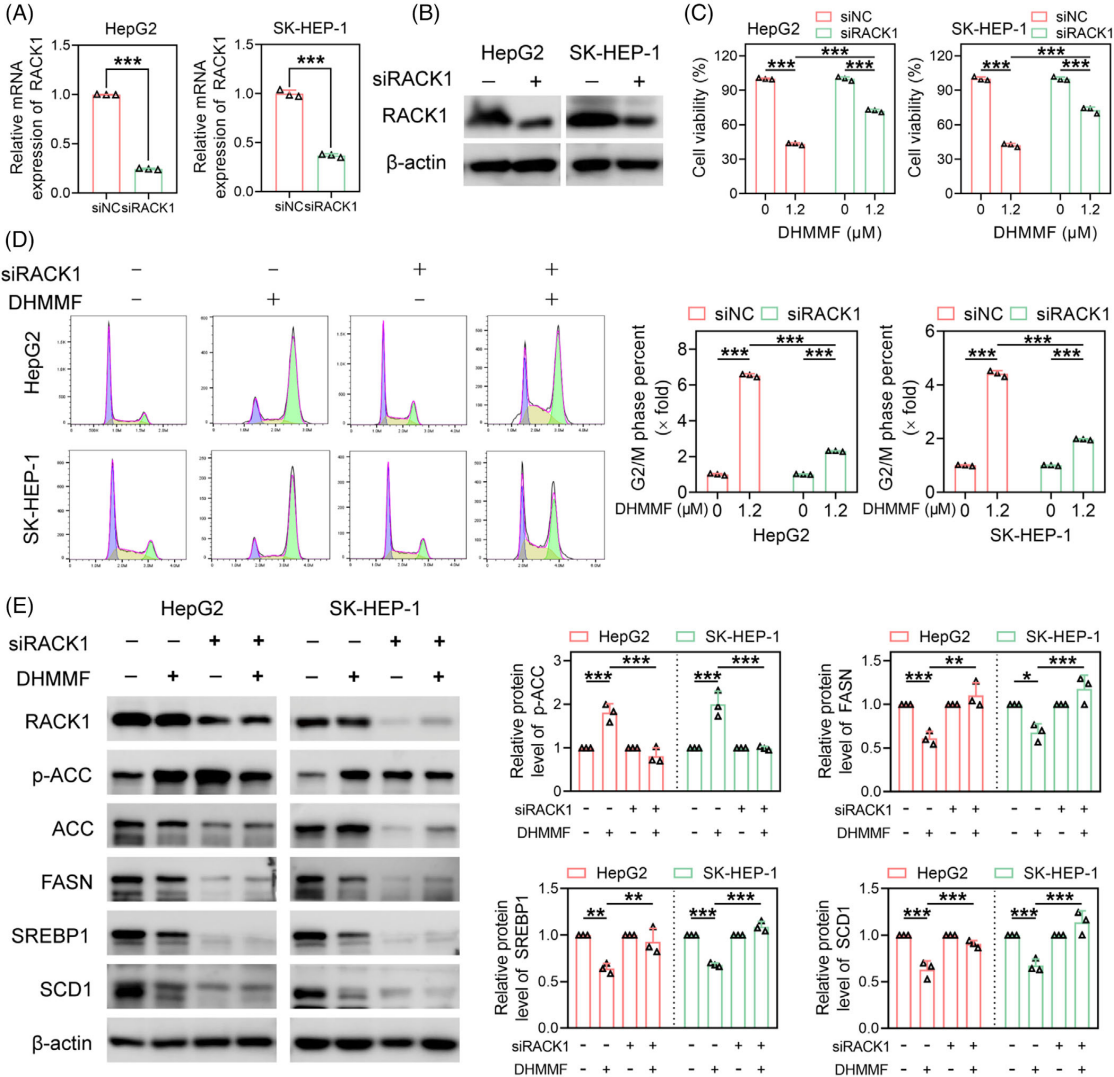
Receptor for activated C kinase 1 (RACK1) is involved in the regulatory effect of (*R*)–7,3′‐dihydroxy‐4′‐methoxy‐8‐methylflavane (DHMMF) on cell cycle arrest and lipid metabolism in human hepatocellular carcinoma (HCC) cells. (A) After the two HCC cell lines were transfected with siNC/siRACK1 sequences for 24 h, RACK1 mRNA levels were detected using quantitative real‐time polymerase chain reaction (qRT‐PCR) (*n* = 3). (B) After the two cell lines were transfected with siNC/siRACK1 sequences for 48 h, RACK1 protein levels were detected using western blotting. (C) After the two cell lines were treated with siNC/siRACK1 for 48 h or with 0 or 1.2 µM DHMMF for 24 h, cell viability was measured using the Cell Counting Kit‐8 assay (*n* = 3). (D) After the two cell lines were treated with siNC/siRACK1 for 48 h and with 0 or 1.2 µM DHMMF for 24 h, flow cytometry was conducted to assess the cell cycle distribution (*n* = 3). (E) After the two cell lines were treated with siNC/siRACK1 for 48 h or with 0 or 1.2 µM DHMMF for 24 h, the expression levels of the proteins related to lipid metabolism were detected using western blotting (*n* = 3). ^**^ *p* < 0.01 and ^***^ *p* < 0.001.

**FIGURE 4:**
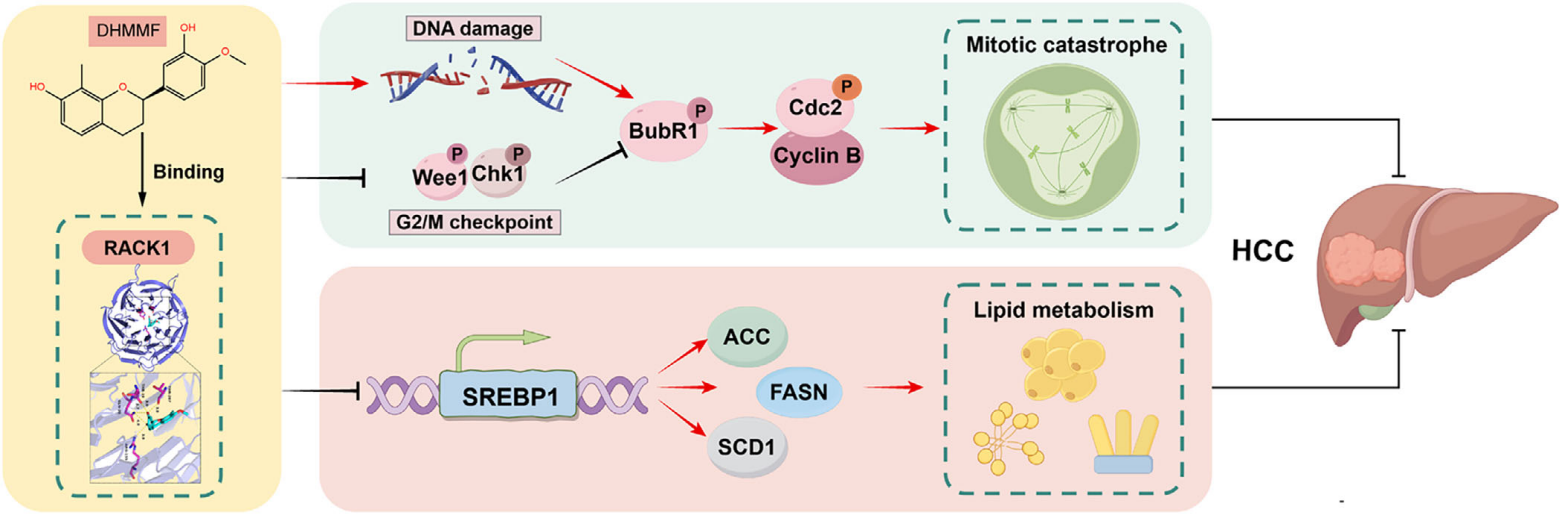
(*R*)–7,3′‐dihydroxy‐4′‐methoxy‐8‐methylflavane (DHMMF) exerted anti‐hepatocellular carcinoma (anti‐HCC) effects by targeting receptor for activated C kinase 1 (RACK1). DHMMF triggered DNA damage in human HCC cells and inhibited G2/M checkpoint protein activation (ChK1 and Wee1), leading to the premature entry of cells with damaged DNA into mitosis. This was followed by the activation of BubR1, downregulation of Cdc2 phosphorylation and upregulation of Cyclin B1 expression, resulting in M‐phase arrest and mitotic catastrophe. Moreover, DHMMF suppressed lipid synthesis in human HCC cells by modulating the function of SREBP1 and its downstream proteins ACC, FASN and SCD1. Additionally, DHMMF directly targeted RACK1, thus influencing cell cycle progression and lipid metabolism and ultimately exerting its anti‐HCC effects.

In summary, this study showed that the natural flavonoid DHMMF induced DNA damage in human HCC cells. DHMMF inhibits Chk1 and Wee1, and thus disrupts the activation of the G2/M checkpoint, causing the DNA‐damaged cells to prematurely enter mitosis, which leads to SAC activation, resulting in M‐phase arrest and mitotic catastrophe in human HCC cells. DHMMF also inhibits lipid synthesis in the human HCC cells by modulating the functions of SREBP1 and its downstream proteins ACC, FASN and SCD1, which contributes to its anti‐HCC effects. RACK1 serves as a direct target of DHMMF, indicating its potential as a therapeutic target in HCC. RACK1 is closely related to cell cycle regulation and can affect the cell cycle of breast cancer cells, thereby promoting their proliferation.^21^ In mouse oocytes, RACK1 interacts with Wee1 to orchestrate the transition between the G2 phase and the M phase by modulating proteins such as Cdc25B, Cdc2 and Cyclin B.^22^ Disruption of RACK1 function can inhibit the Aurora A protein and decrease its phosphorylation, resulting in G2/M phase arrest.^23^ The significance of RACK1 extends to biological metabolic processes as well. In mice, RACK1 plays an important role in glucolipid metabolism, and its absence leads to a reduction in plasma lipid levels.^24^ Hepatocyte‐specific deletion of RACK1 triggers lipid accumulation in the liver.^25^ Additionally, RACK1 collaborates with adiponectin to regulate lipid metabolism.^26^ Therefore, RACK1 is closely associated with DHMMF‐mediated regulation of cell cycle arrest and lipid metabolism in HCC cells. To summarise, DHMMF may exert its anti‐HCC effects through a direct binding to RACK1, thus influencing cell cycle progression and lipid metabolism (Figure 4). Considering that lipids are necessary for mitosis,^27^ DHMMF may affect mitosis in human HCC cells by inhibiting lipid metabolism and reducing the production of fatty acids essential for cell membrane synthesis. This study provides a strong scientific foundation for the future clinical application of DHMMF and offers a novel therapeutic approach for treating HCC.

## AUTHOR CONTRIBUTIONS

**Longyan Wang** and **Peng Tan**: conceptualisation, methodology, data curation, investigation and writing—original draft. **Fei Wang**, **Huiming Huang**, **Xuejiao Wei**, **Zhuguo Wang**, **Xinyu Qiu**, **Yufeng Gao** and **Ruoxin Zhang**: investigation, visualisation and validation. **Pengfei Tu**: formal analysis and methodology. **Zhongdong Hu** and **Jun Li**: conceptualisation, project administration, supervision, funding acquisition and writing—review & editing.

## CONFLICT OF INTEREST STATEMENT

The authors have declared no conflict of interest.

## FUNDING INFORMATION

This study was financially supported by the National Natural Science Foundation of China (82074072, 81873044), the Fundamental Research Funds for the Central Universities (2023‐JYB‐JBQN‐051) and the Talent Cultivation Project of Beijing University of Chinese Medicine (JZPY202206).

## ETHICS STATEMENT

Not applicable.

## Supporting information



Supporting Information

## Data Availability

The data that support the findings of this study are available from the corresponding author upon reasonable request.
