# Supplementary material for: Targeting receptor for activated C kinase 1 with a small molecule induces mitotic catastrophe and suppresses lipid metabolic reprogramming in hepatocellular carcinoma
Source: Clin Transl Med. 2025 Aug 20;15(8):e70460. doi: 10.1002/ctm2.70460 (PMC12367861; doi:10.1002/ctm2.70460)
Supplement: Supplementary file 1 — Supporting Information [file CTM2-15-e70460-s001.docx]

**Supporting Information**

**Targeting RACK1 with small molecule induces mitotic catastrophe and suppresses lipid metabolic reprogramming in hepatocellular carcinoma**

Longyan Wang^a,b,†^, Peng Tan^a,b,†^, Fei Wang^a,b^, Huiming Huang^a,b^, Xuejiao Wei^a,b^, Zhuguo Wang^a,b^, Xinyu Qiu^a,b^, Yufeng Gao^a,b^, Ruoxin Zhang^a,b^, Pengfei Tu^b^, Jun Li^b,*^, and Zhongdong Hu^b,*^

^a^School of Chinese Materia Medica, Beijing University of Chinese Medicine, Beijing 100029, China

^b^Modern Research Center for Traditional Chinese Medicine, Beijing Research Institute of Chinese Medicine, Beijing University of Chinese Medicine, Beijing 100029, China

^†^These authors contributed equally to this work.

^*^Corresponding Author: Zhongdong Hu, No. 11 North 3rd Ring East Road, Chaoyang District, Beijing 100029, China, tel/fax: 8610 6428 6180, e-mail: zdhu@bucm.edu.cn. Jun Li, No. 11 North 3rd Ring East Road, Chaoyang District, Beijing 100029, P. R. China, tel/fax: 8610 6428 6350, e-mail: [drlj666@163.com](mailto:drlj666@163.com).

## 1. Material and methods

**1.1** **Cell culture and drugs**

Human HCC HepG2 and SK-HEP-1 cells were provided by the Cell Center of the Institute of Basic Medical Sciences, Chinese Academy of Medical Sciences, and cultivated in DMEM at 37 °C with 5% CO_2_. (*R*)–7,3’-Dihydroxy-4’-methoxy-8-methylflavane (DHMMF) was obtained from *Resina Draconis* following previous methods^1^, with a purity greater than 99.9%.

**1.2** **Cell viability assay**

The logarithmic-phase HepG2 and SK-HEP-1 cells were digested to form a single-cell suspension and diluted to a concentration of 3 × 10^4^ cells/mL. Then, the cell suspension (100 µL/well) was inoculated in a 96-well plate. After the cells adhered to the plate, the stock solution of 10 mM DHMMF was gradient-diluted to concentrations of 0.2, 0.4, 0.6, 0.8, and 1.0 μM for treatment. At 24, 48, and 72 h after drug treatment, the old medium was aspirated and discarded, and a working solution, which was diluted with CCK-8 reagent (MA0218, Meilun Biotechnology Co., Ltd., Dalian, Liaoning, China) and a basic medium at a 1:10 ratio, was added. After 2 h of incubation, the absorbance values were recorded at 450 nm using a microplate reader.

**1.3** **Flow cytometry analysis for cell cycle detection**

In a six-well plate, 1 × 10^5^ cells were added to each well. Following starvation for 12 h in a basic medium, the cells were treated with the working solution of DHMMF. After 24 h, the cells were harvested, centrifuged, and washed with cold PBS. The samples were subsequently fixed overnight with 70% cold ethanol. The samples were stained with Cell Cycle Detection Kit (550825, BD Biosciences, New Jersey, USA) and incubated for 30 min in the dark. Finally, flow cytometry analysis was performed to assess the effect of DHMMF on the cell cycle of human HCC cells.

**1.4** **Comet assay**

In a six-well plate, 1 × 10^5^ cells were inoculated in each well and subsequently exposed to DHMMF working solution for two days. The assay was performed following the instructions provided with the comet assay kit (KGA1302-100, Jiangsu KeyGen Biotechnology Co., Ltd., Nanjing, Jiangsu, China). The cells were centrifuged to collect them, which were then suspended in PBS to achieve a density of 1 × 10^6^ cells/mL. Preheated normal-melting agarose gel (100 μL) was placed on a microscope slide, mounted on a coverslip, and cooled at 4 °C for 20 min. Then, 75 μL of preheated (70 °C) low-melting agarose gel added to 10 μL of the cell suspension was applied to the normal melting agarose gel on the slide, which was mounted on a coverslip and solidified. The coverslip was removed, and 75 μL of melted low-melting agarose gel was added. The sample was mounted again, after which it was incubated for 30 min to solidify. Next, the coverslip was removed, and the agarose-coated slide was placed in freshly prepared lysis solution (9 mL of lysis buffer mixed with 1 mL of DMSO) for 2 h at 4 °C. The slide was gently rinsed three times with PBS and incubated in a horizontal electrophoresis tank containing alkaline electrophoresis buffer at room temperature for 60 min. Following incubation, electrophoresis was performed at 25 V for 25 min. After electrophoresis, the slide was neutralized three times with PBS for 10 min each. Finally, 20 μL of PI staining solution was added to each slide, which was subsequently stained in the dark for 10 min and visualized using a fluorescence microscope.

**1.5 RNAi**

Both cell lines were inoculated in a 12-well plate and transfected when the growth confluence reached 70–90%. First, combine 100 μL of Opti-MEM medium with 4 μL of siRNAs and gently pipette up and down to ensure thorough mixing. Next, transfer another 100 μL of Opti-MEM medium into an enzyme-free EP tube, aspirate 4 μL of cationic transfection reagent, and gently pipette to blend it uniformly with the medium. After standing at room temperature for 5 min, merge the two mixtures. It is crucial to slowly and gently add the medium containing the cationic transfection reagent dropwise to the Opti-MEM medium with siRNAs. Let the combined mixture stand at room temperature for an additional 15 min. Subsequently, aspirate and discard the spent medium from the 12-well plate, add 800 μL of fresh complete medium, and then introduce the previously mixed 200 μL of Opti-MEM medium into the well. Gently agitate to evenly mix the two media types, and then place the plate in the incubator. Fresh complete medium can be added as needed during the culture period. The transfection process should continue for a minimum of 48 h.The siRNAs were transfected in cells seeded in 12-well plates following the instructions provided. The siRNAs was synthesized by GenePharma (Shanghai, China). Transfection was performed using the GP-transfect-Mate cationic transfection solution (G04026, GenePharma, Shanghai, China). The siRNAs sequences used are presented below:

RACK1: 5’-GCUUCAGCCCUAACCGCUATT-3’;

Negative control (NC): 5’-UUCUCCGAACGUGUCACGUTT-3’.

**1.6 Quantitative real-time PCR (qRT-PCR)**

RNA was extracted from control cells and human HCC cells treated with 1.2 μM DHMMF for 24 h using a Cell RNA Extraction Kit (RC201, Nanjing Vazyme Biotech Co., Ltd., Nanjing, Jiangsu, China). Next, to obtain cDNA, reverse transcription was conducted using TransScript All-in-One First-Strand cDNA Synthesis SuperMix for qPCR (AQ141-01, Beijing TransGen Biotech Co., Ltd., Beijing, China). The cDNA obtained from reverse transcription was amplified using Taq Pro Universal SYBR qPCR Master Mix (Q712-02, Nanjing Vazyme Biotech Co., Ltd.). The specific steps were performed as previously described.^2^ The sequences of primers used are shown below:

RACK1 Forward: CTGCATCTACTCCAAAGTCAG,

RACK1 Reverse: GGTTTGCCAGCATCATGTTTATT;

β-actin Forward: CATGTACGTTGCTATCCAGGC,

β-actin Reverse: CTCCTTAATGTCACGCACGAT.

**1.7 Western blotting**

First, PBS was added to wash the cells treated with DHMMF twice before harvesting the cells using lysis buffer (100 mM DTT, 10 mM Tris, pH 6.8, 2% SDS, and 10% glycerol). Then, western blotting was performed for protein detection following previous methods.^1^ The following antibodies were used in this study: γH2AX (9718, CST, Boston, MA, USA), pHH3 (9701, CST), p-Wee1(Ser642) (4910, CST), p-Chk1(Ser317) (12302, CST), p-Cdc2(Tyr15) (4539, CST), Cyclin B1 (12231, CST), ACC (3676, CST), p-ACC (11818, CST), α-tubulin (11224–1-AP, Proteintech GroupWuhan, Hubei, China), FASN (10624–2-AP, Proteintech), β-actin (sc-58673, Santa Cruz Biotechnology, Santa Cruz, USA), Chk1 (sc-8408, Santa Cruz Biotechnology), and Cdc2 (sc-54, Santa Cruz Biotechnology), p-BubR1 (S670, Abcam, Cambridge, England), BubR1 (A01564-1, Boster Biological Technology Co., Ltd, Wuhan, Hubei, China).

**1.8 Immunofluorescence assay**

Both cell lines (1 × 10^5^ cells/dish) were seeded in laser confocal dishes. After the cells adhered to the dish, they were treated with a DHMMF working solution for 24 h, fixed for 1 h with paraformaldehyde at 4 °C, and then rinsed. The cells were subsequently blocked and permeabilized with a 1% PBS-BSA solution containing 0.1% Triton X-100 at ambient temperature for 45 min. Finally, the cells were incubated with α-tubulin (1:500) at 4 °C overnight. Following incubation, the α-tubulin solution was discarded, and the cells were incubated with secondary antibodies for 60 min in the dark at ambient temperature. After incubation, a DAPI solution was added, and a drop of glycerol was used to mount the coverslip. The cells were examined, and photographs were taken with an Olympus FV1000 confocal laser scanning microscope (Olympus, Tokyo, Japan).

**1.9** **Untargeted metabolomics**

Initially, HepG2 cells were inoculated in 10-cm dishes at a density of 2 × 10^6^ cells/dish. After incubating for 24 h, the old medium was discarded. The cells in the treatment group were exposed to a complete medium containing 1.2 μM DHMMF, while those in the control group were exposed to a blank complete medium. After 24 h, the cells were collected, and extraction was performed using an extraction reagent (methanol:acetonitrile:water = 2:2:1, containing an isotope-labeled endogenous reference mixture). This mixture was vortexed for uniform mixing, frozen and thawed in liquid nitrogen, and then centrifuged to obtain the supernatant for instrumental detection. Additionally, the supernatants of all samples were mixed to prepare quality control (QC) samples for instrumental detection. An LC-MS equipment (Vanquish, Thermo Fisher Scientific, UPLC; Thermo Fisher Scientific, Orbitrap Exploris 120) was used to analyze the samples. The chromatographic column used was ACQUITY UPLC BEH Amide (1.7 μm, 2.1 mm*50 mm). The injection volume was 2 μL, and the autosampler temperature was 4 °C. Mobile phase A was 25 mmol/L H_2_O + 25 mmol/L HCOOH, and mobile phase B was 100% acetonitrile (CAN). The results were processed and analyzed accordingly.

**1.10** **Measurement of Triglycerides**

Both cell lines were seeded in 10-cm dishes at 2 × 10^6^ cells/dish. After treatment with DHMMF for 24 h, the cells were collected in 2 mL Eppendorf tubes and centrifuged for 5 min at 4 °C and 300 rcf. Then, the cells were washed three times with precooled PBS, and an appropriate amount of RIPA lysis buffer was added to lyse the cells by repeated pipetting. The mixed sample was placed on ice for 30 min to perform cell lysis. After 10 min of centrifugation at 4 °C and 10,000 rcf, the supernatants were collected. Following the instructions of the Triglyceride Assay Kit (A110-1-1, Nanjing Jiancheng Bioengineering Institute, Nanjing, Jiangsu, China), the corresponding working solution system was prepared in a 96-well plate. The plate was shaken to mix the solution evenly, followed by 10 min of incubation at 37 °C. The absorbance was measured at 500 nm using a microplate reader.

**1.11 Pull-down mass spectrometry**

HepG2 cells were cultivated in a 10 cm dish until they reached about 80% confluence, after which the cells were scraped into an EP tube. Lysis buffer was added to the EP tube to lyse the cells at a low temperature. After centrifugation, the supernatant was aspirated and stored at 4 °C for later use. Additionally, a portion of the supernatant was collected and diluted to determine its concentration using the BCA method. First, 50 μL of the resuspended gel was added to a spin column, and 250 μL of TBS solution was subsequently added. The spin column was centrifuged at 1300 ×*g* for 60 s. Second, a mixture containing 300 μL of biotin-labeled DHMMF and biotin (1 μg/μL) was introduced into the spin column, which was gently shaken on a rotating platform and incubated at 4 °C. After incubation, the column underwent 60 s of centrifugation again at 1300 ×*g*. Next, a biotin-blocking solution (250 μL) was added to the spin column. The column was cultured for 5 min at ambient temperature and subsequently centrifuged at 1300 ×*g* for 60 s. Next, 300 μL of protein mixture (containing 1 mg of protein) was added to the spin column. The column was gently shaken on a rotating platform and incubated at 4 °C. After incubation, the column was centrifuged for 60 s at 1300 ×*g*. Next, 250 μL of wash buffer was added to the spin column, followed by 1 min of incubation at ambient temperature. Finally, the samples were centrifuged for 60 s at 1300 ×*g*, after which the beads were collected.

**1.12 Pull-down immunoblotting**

Classifying 350 μL of streptavidin magnetic beads (22308-1, Suzhou BeaverBeads, suzhou, Jiangsu, China) into three groups: DHMMF-biotin, competitive experiment, and control groups. The first two groups were incubated with an appropriate amount of DHMMF conjugated with biotin, while the control group was treated with the same amount of DMSO. The cell lysates were collected and divided into the same three groups as the magnetic beads. In the competitive experiment group, an appropriate amount of DHMMF was added to bind with the target protein. An equal volume of DMSO was added to the other two groups, which were subsequently incubated at a low temperature. The lysates from the same group were added to the corresponding magnetic beads and mixed by inverting the tubes to allow binding. After incubation, the magnetic beads were carefully washed. The beads were boiled to elute the bound proteins, which were collected. Finally, western blotting experiments were performed on the collected proteins.

**1.13 Cellular thermal shift assay (CETSA)**

Human HCC cells were inoculated in two 10-cm dishes containing the same number of cells. After the cells adhered to the dishes, one dish was treated with DHMMF, and the other dish served as the control group. After 2 h of incubation, the cells were harvested and lysed, and the supernatants were collected. The two groups of proteins obtained above were each divided into nine aliquots of 100 μL and placed in 200 μL EP tubes. Gradient heating was performed on a PCR machine, starting at 46 °C and increasing to 64 °C. After heating, the samples were rapidly frozen using liquid nitrogen and thawed in a 37 °C water bath; this cycle was performed three times. The supernatants were collected by centrifugation, with the addition of 5× loading buffer. The samples were pooled and used for western blotting experiments.

**1.14 Drug affinity responsive target stability (DARTS)**

Human HCC cells were inoculated in 10-cm dishes and allowed to grow until they reached 90% confluence. Then, proteins were extracted with IP lysis buffer, and the protein content was determined. The cell lysate was divided equally into four groups: the control, control + enzyme, drug, and drug + enzyme groups. Appropriate amounts of DHMMF were added to the drug group and the drug + enzyme group, while the same amount of DMSO was added to the control and control + enzyme groups. A certain volume of 10× TNC solution was added to all four tubes of protein, which were placed on a shaker and incubated at 4 °C for 4 h. The protein content in each tube was calculated based on the measured protein concentration. The protease stock solution was diluted with 1 × TNC solution and added to the control + enzyme group and the drug + enzyme group such that the ratio of enzyme to protein was 1:1000. After incubation for 30 min to allow enzymatic digestion, a certain volume of 5× loading buffer was added to all four tubes of protein. Finally, the proteins were collected and used for western blotting experiments.

**1.15 Surface plasmon resonance (SPR)**

A Biacore T200 instrument (GE Healthcare Life Sciences, Pittsburgh, USA) was used to evaluate the binding kinetics between DHMMF and RACK1. The recombinant human RACK1 protein (Abcam) was diluted with sodium acetate (pH 4.0) to 50 μg/mL and immobilized onto the chip surface at 10 μL/min. The coupling level was set to 9000 to generate a coupling plot. A blank surface underwent a similar treatment but without any protein solution and served as the reference surface. Each analyte was diluted to several concentrations in a 96-well plate, followed by injection over the target protein-coupled chip from low to high concentrations. The flow rate was maintained at 30 μL/min for 150 s. After injecting the analytes at all concentrations, chip regeneration was performed with 10 mM glycine hydrochloride (pH 2.0) solution for 5 min. This process was repeated until all corresponding concentrations of the analytes were tested. The data obtained from the samples were collected using the BIAcore T200 Control software (v.2.0, GE Healthcare), while those from the reference channel were subtracted. The data were globally fitted to the 1:1 Langmuir binding model using the BIAcore T200 Evaluation Software (v.2.0, GE Healthcare) to obtain the binding and dissociation constants.

**1.16 Molecular docking analysis**

To create a 2D structural formula of DHMMF, it was first processed with AutoDock by adding hydrogen atoms and making other necessary modifications and then output as a ligand file in the PDBQT format. The protein structure of RACK1 (4AOW) was obtained from the PDB website (https://www.rcsb.org/). Using PyMOL, water molecules and ligand residues in the protein receptor were removed. Then, the modified protein was imported into AutoDock for further processing, such as adding hydrogen atoms and calculating charges to convert it into a receptor. AutoDock Tools were used to simulate docking between the receptor and the ligand. The generated results were imported into PyMOL, and the docking mode with the highest binding energy was selected to perform visual analysis.

**1.17 Statistical analysis**

All data were statistically analyzed using the GraphPad Prism 9.3 software, and the results are presented as the mean ± SD. Between-group differences were evaluated by a two-tailed Student’s *t*-test, whereas among-group differences were determined by two-way ANOVA. All differences among and between groups were considered to be statistically significant at *P* < 0.05.

**Reference**

1 Tian Y, Wang L, Chen X, Zhao Y, Yang A, Huang H, et al. DHMMF, a natural flavonoid from Resina Draconis, inhibits hepatocellular carcinoma progression via inducing apoptosis and G2/M phase arrest mediated by DNA damage-driven upregulation of p21*.* *Biochemical Pharmacology* 2023;**211**:115518.

2 Hu Z, Wang Y, Huang F, Chen R, Li C, Wang F, et al. Brain-expressed X-linked 2 Is Pivotal for Hyperactive Mechanistic Target of Rapamycin (mTOR)-mediated Tumorigenesis*.* *The Journal of biological chemistry* 2015;**290**:25756-65.


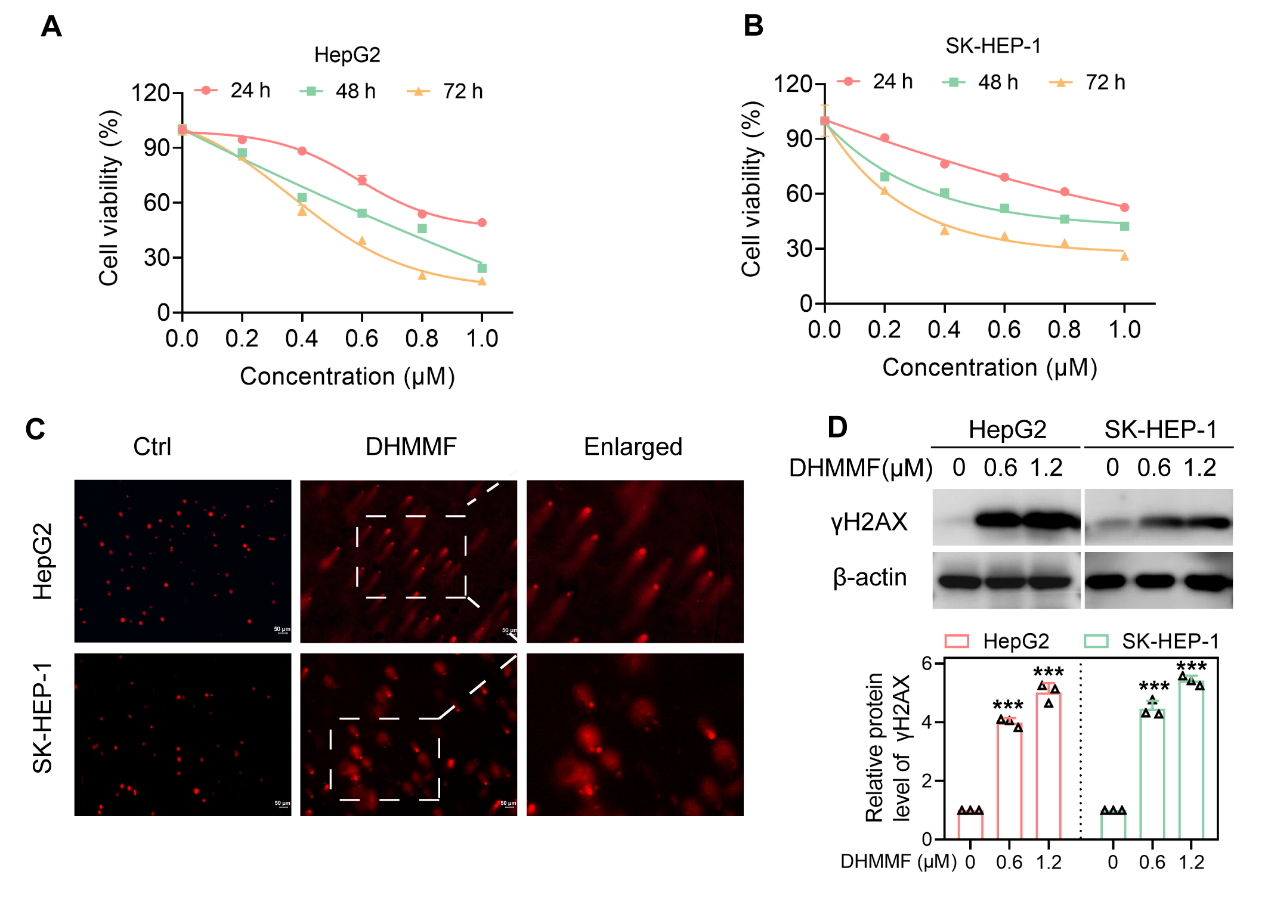


**Figure S1. DHMMF suppressed proliferation and induced DNA damage in human HCC cells.** (A and B) HepG2 and SK-HEP-1 cells were treated with DHMMF at concentrations of 0, 0.2, 0.4, 0.6, 0.8, and 1.0 μM. Cell viability was assessed using the CCK8 assay at 24, 48, and 72 h (n=3). (C) After treating HepG2 and SK-HEP-1 cells with 0 and 1.2 μM DHMMF for 24 h, the DNA damage in the cells was evaluated using the comet assay (n=3). (D) Following treatment of human hepatocellular carcinoma HepG2 and SK-HEP-1 cells with 0, 0.6, and 1.2 μM DHMMF for 24 h, the protein expression level of γ-H2AX was detected using western blotting (n=3). ^***^*P* < 0.001.


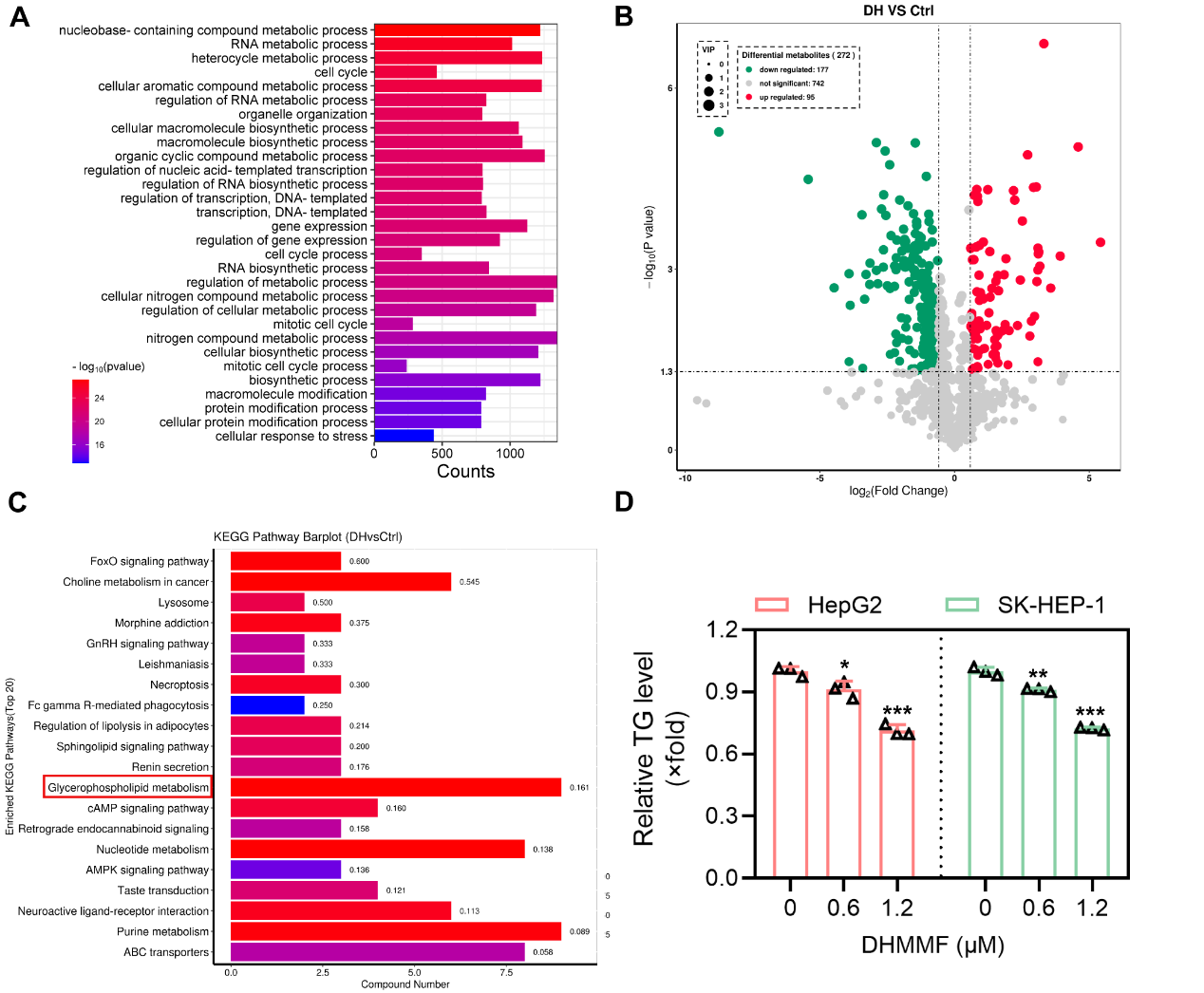


**Figure S2. Effects of DHMMF on lipid metabolism in human HCC cells.** (A) GO enrichment analysis of the transcriptome revealed the regulatory effects of DHMMF treatment on biological processes in human HCC cells, with red indicating increased confidence and blue indicating decreased confidence. (B) Non-targeted metabolomics analysis of the metabolic changes in human HCC cells before and after DHMMF treatment. (C) The altered metabolites in human HCC cells after DHMMF treatment were enriched mainly in the glycerophospholipid metabolism pathway. (D) After treating human hepatocellular carcinoma HepG2 and SK-HEP-1 cells with 0, 0.6, and 1.2 μM DHMMF for 24 h, the triglyceride (TG) content was measured using a kit (n=3). ^*^*P* < 0.1, ^**^*P* < 0.01, and ^***^*P* < 0.001.


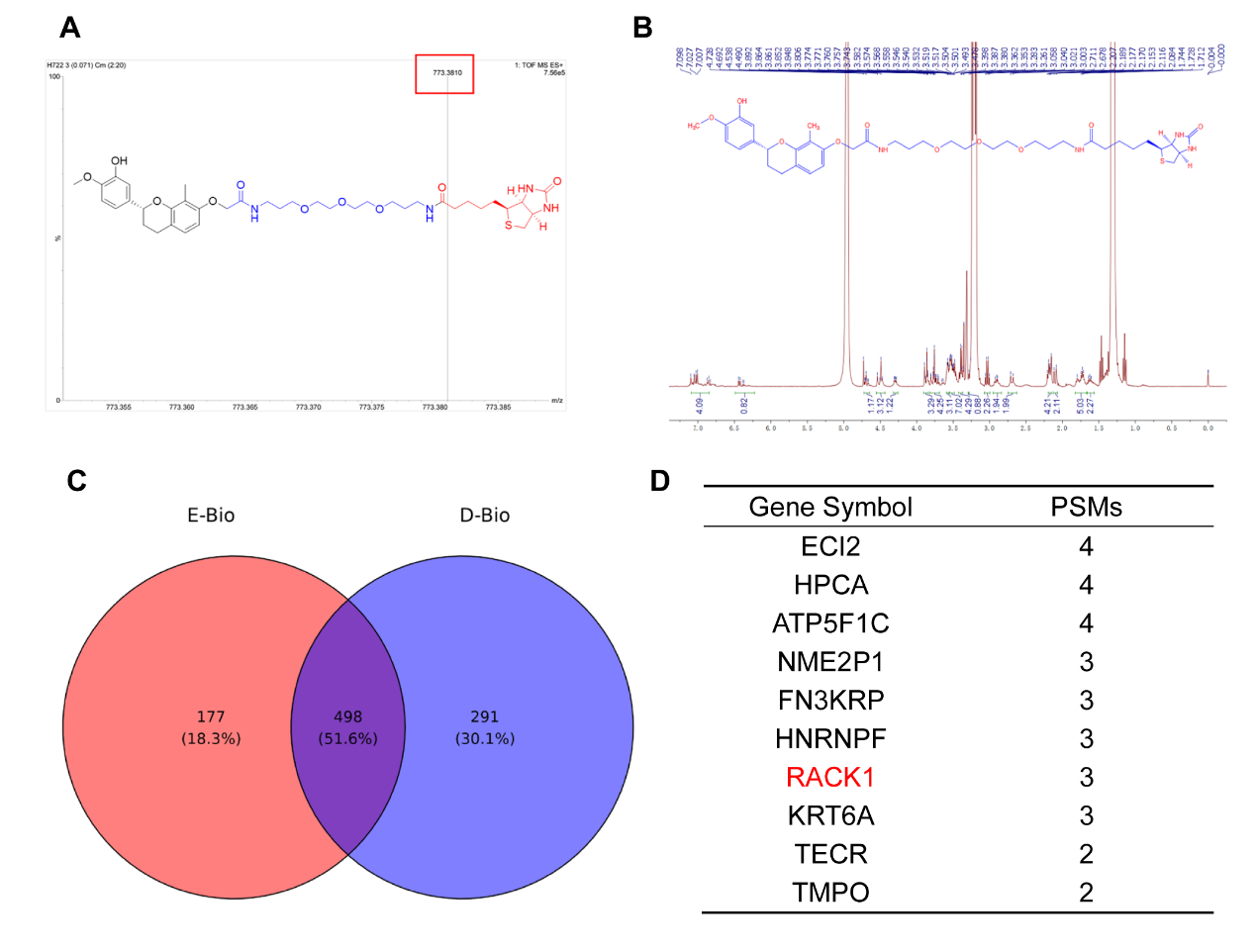


**Figure S3. RACK1 is the direct target of DHMMF.** (A) The mass spectrometry data of the DHMMF biotin probe. (B) The NMR data of the DHMMF biotin probe. (C) Differential analysis of proteins captured between the biotin-labeled DHMMF group (E-Bio) and the biotin-only control group (D-Bio). (D) Top 10 high-confidence candidate proteins identified through reliability scoring.
